# Effect of functionalization on the adsorption performance of carbon nanotube as a drug delivery system for imatinib: molecular simulation study

**DOI:** 10.1186/s13065-024-01197-0

**Published:** 2024-04-27

**Authors:** Masume Rezazade, Sepideh Ketabi, Mahnaz Qomi

**Affiliations:** 1 Department of Medicinal Chemistry, Faculty of Pharmacy, Tehran Medical Sciences, Islamic Azad University, Tehran, Iran; 2 Department of Chemistry, Faculty of Pharmaceutical Chemistry, Tehran Medical Sciences, Islamic Azad University, Tehran, Iran; 3 Active Pharmaceutical Ingredients Research (APIRC), Tehran Medical Sciences, Islamic Azad University, Tehran, Iran

**Keywords:** Imatinib, Solvation, Association free energy, DFT, Monte Carlo simulation

## Abstract

In this study, efficiency of functionalized carbon nanotube as a potential delivery system for imatinib anti-cancer drug was investigated. Accordingly, carboxyl and hydroxyl functionalized carbon nanotube were inspected as a notable candidate for the carriage of this drug in aqueous media. For this purpose, possible interactions of imatinib with pure and functionalized carbon nanotube were considered in aqueous media. The compounds were optimized in gas phase using density functional calculations. Solvation free energies and association free energies of the optimized structures were then studied by Monte Carlo simulation and perturbation method in water environment. Outcomes of quantum mechanical calculations presented that pure and functionalized carbon nanotubes can act as imatinib drug adsorbents in gas phase. However, results of association free energy calculations in aqueous solution indicated that only carboxyl and hydroxyl functionalized carbon nanotubes could interact with imatinib. Monte Carlo simulation results revealed that electrostatic interactions play a vital role in the intermolecular interaction energies after binding of drug and nanotube in aqueous solution. Computed solvation free energies in water showed that the interactions with functionalized carbon nanotubes significantly enhance the solubility of imatinib, which could improve its in vivo bioavailability.

## Introduction

Regardless of the medicinal advances, even now cancer is the leading cause of death. Therefore, investigating anticancer drugs and reducing their side effects is the main goal of much recent research. Many Tyrosine kinase inhibitors, such as Imatinib (IMA), are extensively utilized in the clinical setting for the treatment of various types of cancers. This is primarily due to the well-established association between tyrosine kinase-related pathways and tumor metastasis as well as angiogenesis [1].

The mechanism of action of IMA involves the prevention of tyrosine kinase receptor phosphorylation. By binding to the ATP site of these receptors, IMA effectively inhibits the proliferation and persistence of cancer cells [2, 3]. Numerous studies have demonstrated the efficacy of IMA in treating different types of cancers, including chronic myeloid leukemia and gastrointestinal stromal tumor [2, 4, 5]. Notably, IMA functions as an anti-proliferative agent, interfering with the proliferation of cancer cells [6]. The utilization of IMA has been authorized as an adjunctive treatment for patients with gastrointestinal stromal tumor who are at a significant risk of relapse and require long-term medication. In these patients, it is of utmost importance to highlight the criticality of administering optimal doses of the drug to prevent low plasma IMA levels [7].

Periorbital edema and epiphora have been identified by Fraunfelder et al. as two commonly observed ocular side-effects associated with the use of IMA [8]. Other side effects of IMA encompass nausea, vomiting, diarrhea, skin rash, musculoskeletal complaints, fatigue, hemorrhage, edema, hand-foot skin reaction, skin and hair discoloration, mucositis, hypertension, cardiac toxicity, hypothyroidism, liver transaminase changes, and hematological abnormalities [7].

To mitigate the side effects or manage optimal drug levels during long-term treatments, various drug delivery systems, including nanocarriers, have been implemented. These nanostructures offer several advantages, such as a high specific surface area, chemical activity, and increased permeability [9, 10]. Notably, nanotubes have the ability to penetrate cells and even reach the cell nuclei [11–16]. Furthermore, their non-toxic properties make them promising candidates for biomedical applications [17, 18].

Due to the high surface area of Carbon nanotubes (CNTs), they are excellent candidate for medicinal chemistry applications. CNTs can be applied as an appropriate carrier for brain delivery due to their small size and hydrophobic nature, which helps in better cellular internalization [19]. Crossing the barrier of blood–brain is also an important application of CNTs that is utilized in different drug deliveries, as many drugs suffer from an inability to reach tumors (the blood–brain barrier prevents drugs from destroying brain tumors [20, 21]. Moreover, CNTs have been immensely used now for neural regeneration due to their conductive nature.

CNTs are chemically inappropriate with many organic and inorganic solvents; therefore, a major disadvantage for the application of CNTs in nanomedicine is that CNT solutions are difficult to access. The hydrophobic nature of CNTs, and the powerful interactions between the distinct tubes induce CNT aggregation and decrease their solubility. Therefore, non-functionalized CNTs are poorly soluble in water [22]. Nevertheless, modifications of their structures using different functional groups can overwhelmed this drawback and improve biocompatibility [19]. functionalization of the surface and alteration of the properties of carbon nanotubes (CNTs) have been explored as a promising approach to reduce their toxicity[23]. The functionalization of CNTs establishes them as essential carriers for delivering anticancer drugs and genes, resulting in reduced side effects [24]. By functionalization CNTs with polymers, metal nanoparticles, and biomaterials, the properties of CNTs can be altered, thereby enhancing their usage in various fields [25]. The development of CNT-based drug carrier systems for the delivery of anti-cancer drugs has demonstrated that different types of functionalization can modify the properties of CNTs both in vitro and in vivo [26].

Research has shown that chemically functionalizing CNTs with oxygenated functional groups, such as carboxylic acid, ketone, alcohol, and ester groups, provides the advantage of good solubility in various organic solvents and aqueous solutions. This advantage arises due to the presence of different functional groups on carbon nanotubes with either polar or non-polar properties. Additionally, these functionalized CNTs exhibit stability and can physically absorb drugs through hydrogen bonding, making them desirable for drug delivery systems [27]. In a study conducted by Taghavi et al., polyethyleneimine grafted CNTs were used as vectors or delivering shRNA with covalently attached aptamer, for the delivery of doxorubicin used in the treatment of human gastric cells. The targeted delivery showed strong selectivity and inhibited the growth of gastric cancer cells [28]. Functionalization of CNTs with amino groups of proteins enables CNTs to permeate biological lipid membranes through either an active mechanism or passive diffusion [29].

Two main strategies for functionalization of CNTs have been identified: covalent and non-covalent functionalization. Covalent functionalization, such as through the addition of carboxylic acid or hydroxyl groups, has proven to be advantageous and easily controllable, making it the focus of investigation for improving the use of CNTs as drug delivery systems [30]. Moreover, the presence of COOH, OH, NH_2_, and other functional groups on the CNTs' surface has been shown to alter their characteristics and functionality, including surface area, hydrophilicity, and drug adsorption capabilities [31, 32].

Functionalization through COOH and OH groups can also impact solubility, biocompatibility, selectivity, blood circulation time, and retention in tumors, making CNTs more suitable for various applications [33]. Experimental and computational studies have investigated the adsorption of 5-FU onto covalently functionalized CNTs with carboxyl and hydroxyl groups as a function of pH, providing insights into the optimal conditions for drug loading and release [34]. Magnetic solid-phase extraction techniques using COOH-MWCNTs with magnetic nanoparticles have also been explored for the extraction of drugs from human plasma samples [35]. Stable binding and potential for drug delivery have also been demonstrated in studies investigating the interaction of Remdesivir [36] and 5-aminolevulinic acid anticancer drug [37] on COOH-functionalized CNTs. Furthermore, a nanocomposite of CNT-COOH/MnO_2_/Fe_3_O_4_ has exhibited a high adsorption capacity for drugs such as ibuprofen and paracetamol from aqueous solutions [38]. Notably, covalent functionalization, including COOH functionalization, has been found to enhance the loading capacity of the drug doxorubicin [39].

Density functional calculations comparing the adsorption of doxorubicin on non-functionalized CNTs, hydroxyl-modified CNTs, and carboxylic acid-modified CNTs in gas and solution phases have indicated no significant difference in the distance between doxorubicin and CNTs before and after adsorption, which determines the stability of the complexes [40]. The obtained negative adsorption energies have shown the favorable interactions of doxorubicin and CNT. In another research, molecular dynamics simulations have been applied to investigate the adsorption of Paclitaxel on the pure, Carboxyl, and hydroxyl functionalized CNT. The results have revealed that the presence of functional groups on CNT increases drug adsorption [41].

Earlier studies have indicated that the carbon atoms in CNTs display chemical reactivity towards numerous substances. Furthermore, functionalization has the potential to reduce the cytotoxicity of CNTs and enhance their solubility [42]. Given the aforementioned research demonstrating that the functionalization of carbon nanotubes enhances their solubility and biocompatibility, making them suitable for drug delivery applications, this research study aimed to investigate the impact of COOH and OH functionalization on the adsorption performance of CNTs as a drug carrier for IMA. The adsorption of IMA on non-functionalized CNT was compared to carboxyl and hydroxyl functionalized CNT in both gas phase and aqueous media. The possible bindings were examined in the gas phase using density functional analysis. Subsequently, the interactions between IMA and CNTs in an aqueous solution were investigated, and the solvation free energies of the IMA-CNTs complexes were determined using Monte Carlo simulation.

## Computational method

This research focused on studying the proficiency of CNT and functionalized CNT in transporting the IMA molecule and the solubility of its compounds in an aqueous media. The critical concern of this research was to investigate the interaction between the drug (IMA) and functionalized CNT in a water solution, and to determine the binding energies and hydration of their structures. Computational chemistry offers a practical and cost-effective approach for optimizing several nanocarriers and provides an excellent opportunity to effectively simulate their interactions with desired drugs. Additionally, the possibility of using other materials, such as gold clusters, as carriers for thioguanine [43], glutamate [44], and 5-fluorouracil [45] in an aqueous solution has also been studied using quantum mechanics and molecular simulation.

This research encompassed two parts: Quantum Mechanical (QM) and Molecular Mechanical calculation (MM). In the QM part, the structures were optimized in the gas phase, and then the corresponding binding energies were computed. In the second part, MC was used to determine the solvation free energies and association free energies of the compounds in an aqueous solution. This approach, integrating QM and MM methods, demonstrates acceptable precision while maintaining efficient computational speed. The examination of drug-carrier interactions is conducted through a QM method, and the resulting optimized quantum mechanical structures are placed within a realistic solvent environment. In QM calculations for solution studies, a common practice involves utilizing a solvent model like the polarizable continuum model (PCM), where the solvent is represented as a continuous medium with a dielectric constant (ɛ) rather than individual solvent molecules. In fact, in the gas phase QM calculations, the molecules were considered as isolated species, and the structure’s stability and binding energies were evaluated. However, a drug–water potential energy surface cannot be constructed from accurate QM calculations, even with recent growth in computer power, because too many points are required. While the study of the interactions in aqueous solution by QM method is performed using the QM solvent model methods for the solution phase, it could not use all the solvent molecules in calculations. The construction of a potential energy surface for such large systems, using more elaborate QM techniques, is not currently feasible. Thus, it is suitable to use computer simulation methods for these systems. The accurate computation of solvation free energies and association free energies in solvents through molecular simulations represents a significant advancement in computational chemistry, widely applied in investigations of solvation and binding free energies.

The interaction between the solute and solvent molecules plays a crucial role in understanding the various molecular processes involved in chemistry. Energy minimization by QM calculations was used to prepare the system for MC simulation in solution to relieve any unfavorable interactions in the initial configuration. The density functional theory (DFT) method was applied in QM calculations which is good compromise between computational cost, coverage, and accuracy of results [46]. Takatani et al. [47] tested MP2, SCS-MP2, SCS-CCSD, SCS(MI)-MP2 and B2PLYP methods against CCSD(T) for the small model complexes. They found that the density functional method outperforms all other methods.

The lowest-energy structures (IMA, CNTs, and complexes) after geometry optimization were imported into further MC simulation. Since the purpose of this work is the comparison of the solvation free energies and association free energies in aqueous solution, it was considered all the interactions of water molecules with solute in the solution phase. The goal of each simulation procedure is the simplicity of the system together with the quality of results. The interactions of solutes and solvent molecules were analyzed by only considering intermolecular potential functions. This procedure allowed us to generate the low-energy configurations at a minimal computational cost. The method emerged as a good compromise between computational cost, coverage, and accuracy of results.

### QM

**Fig. 1:**
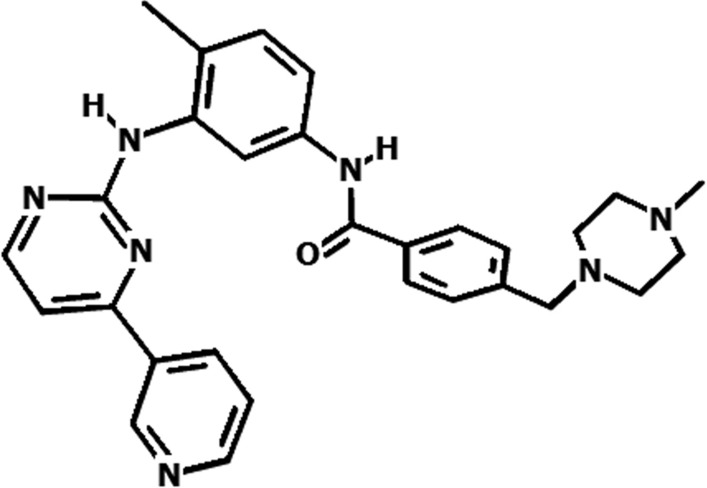
Structure of IMA

In this study, a Zigzag (9, 0) CNT with 180 atoms and a length of 17.08 Å and radius of 3.61 Å, as well as two functionalized CNTs (OH and COOH functionalized) were evaluated as drug carriers for IMA. The structure of IMA has been shown in Fig. 1.

The interactions of IMA and functionalized nanotube were considered from C=O group and pyridine ring. The NH sites did not prefer to interact with CNT due to steric hindrance.

Optimization of CNT, OH– and COOH-functionalized CNTs, drug (IMA), drug-functionalized CNT and drug-non functionalized CNT complexes was optimized by DFT calculation using wB97XD functional [48, 49] method and 6-31G++ (d,p) [50, 51] basis set.

The assessment of the performance of different functional combined with various large basis sets for the computation of interaction energies that involve hydrogen bonding [52] has proposed functional/basis set combinations in a sequence of increasing size and expense to deliver acceptable precision for large systems without an excessive computational load. Given that hydrogen bonding was anticipated to be the principal interaction in our investigation, it is advisable and suitable [52, 53] to employ the 6-31G++ (d,p) basis set with the B3LYP and wB97XD functional, as it results in a suitable balance between precision and efficiency for the study of extensive systems. Consequently, the employed basis set was 6-31G++ (d,p), which encompasses supplementary diffuse functions on oxygen, nitrogen, and hydrogen.

In large-scale systems such as biomolecules [54, 55] and nanostructured materials [56, 57], the stability and reactivity are significantly influenced by weak and long-range van der Waals interactions. The importance of van der Waals interactions results from the large surface areas of interaction due to the delocalization and dispersion effects. Conventional hybrid DFT functionals have a fundamental limitation in considering the dispersion interaction energy in weak interactions [58–60]. The Grimme’s DFT-D2 functional [48] introduces an additional term to the DFT total energy to account for dispersion forces. The wB97XD functional incorporates a version of Grimme’s D2 dispersion model, which includes empirical dispersion, and enables accurate quantum mechanical calculations for nanosized systems [49].

Vibrational analysis was conducted on the optimized compounds, and the characteristics of stationary points were examined for IMA, CNT, and IMA-CNT complexes. The absence of imaginary vibration frequency in the calculations confirms the stability of the structures. The QM computations were carried out using the GAMESS-US quantum chemistry package [61].

The optimized structures of IMA, CNTs, and CNTs-IMA complexes, along with the atomic partial charges obtained from the QM part, were utilized in the MC simulation. Diluted aqueous solutions of these complexes were modeled for the simulation.

### MC

This part of the study aimed to investigate the possibility of interaction between IMA and CNT, as well as functionalized CNTs, in aqueous media. In MC simulations, Canonical ensemble and Metropolis sampling [62] were employed.

The sample size of the system affects the accuracy of Monte Carlo calculations. This factor is due to locating a limited number of molecules in a box followed by the subsequent application of periodic boundary conditions, which introduces an error in the molecular correlations. Periodic boundary conditions enable a simulation to be performed using a relatively small number of particles, in such a way that the particles experience forces as if they were in the bulk fluid. However, the limitation of the periodic cell is that for a given system, the error decreases with the sample size. In most cases of interest, we do not know how to choose the size of the system to minimize the effect of periodic boundary conditions. The range of the interactions is essential and, if the cell size is large compared to the range over which the interactions act, then there should be no problem. For the relatively short-range Lennard–Jones potential, the cell should have a side greater than approximately 6σ [63]. For long-range electrostatic interactions, it is often necessary to accept that some long-range order with be imposed upon the system. The most straightforward test is to perform a series of calculations in which the sample size is systematically increased until the calculated values remain unchanged. In this research, the optimum edges of the box were 50 × 50 × 50 A˚^3^, which corresponds to almost 4000 H_2_O molecules with a density of 0.993 g/cm^3^ at 298 K, [64, 65]. Dilute solutions of the IMA and IMA-CNT complexes were implemented. In fact, one molecule of solute was placed within the cubic box of water, and then, depending on the size of the solute (IMA, CNTs, and IMA-CNTs complexes), some water molecules were removed from the box. The number of added water molecules varied due to the different sizes of the solutes.

In each simulation cycle, a randomly selected H_2_O molecule was translated by ± 0.13 Å and rotated by ± 10°, utilizing periodic boundary conditions. The generated configurations were accepted with a 50% probability. Each run comprised 107 configurations, which were extended to minimize statistical error. The total potential energy of a solution includes intermolecular interactions (solvent–solvent and solute–solvent interactions) and intramolecular interaction energies. Intermolecular energies encompass van der Waals and electrostatic energies.

To compute the intermolecular interaction energies between H_2_O molecules, the Transferable Intermolecular Potential function (TIP3) was utilized [66, 67]. This function incorporates parameters such as q, A, and C to account for the electrostatic, repulsive, and attractive van der Waals interactions. To determine the interaction energies between the solute and H2O, the Lennard–Jones (LJ) potential, characterized by parameters ε and σ, was employed, along with the Coulomb potential for electrostatic interactions, characterized by parameter q. Appropriate Lennard–Jones parameters for atoms in CNT [68] and IMA [69] were applied in the computations. The site–site LJ parameters were derived using the Lorenz-Berthelot combining rule [70]. As previously mentioned, the atomic charges, q, were computed in the preceding QM section.

Free energy differences were calculated by Free Energy Perturbation (FEP) theory using Zwanzig equation [71]:

1 $$\Delta {{\text{G}}_{(\text{A}\to \text{B})}}=-\text{RT Ln}<\exp -({{\text{E}}_{\text{B}}}-{{\text{E}}_{\text{A}}})/\text{RT}{{>}_{\text{A}}}$$

The solvation free energy (ΔGsol(A)) refers to the thermodynamic change in free energy that occurs when a species is transferred from a gaseous state to a solution. Practically, the solvation free energy is determined through a perturbation method, where the species vanishes in both the gas and solution phases. The solvation free energy (ΔGsol(A)) refers to the thermodynamic change in free energy that occurs when a species is transferred from a gaseous state to a solution. Practically, the solvation free energy is determined through a perturbation method, where the species vanishes in both the gas and solution phases:

2 $${\Delta }\text{G}_{{_{{\text{sol(A)}}} }} = {\Delta }\text{G}_{{_{{{\text{gas(A}\to 0)}}} }} - \Delta {\text{G}}_{{{\text{sol(A}\to 0})}}$$

Association free energies of the interaction of IMA and CNTs were calculated using the appropriate thermodynamic cycle. By considering Eq. 2 and the thermodynamic cycle, aassociation free energies (∆G_ass(IMA -CNT))_ were calculated by:

3 $${\Delta }\text{G}_{{{\text{ass(IMA - CNT}})}} = {\Delta }\text{G}_{{{\text{sol(IMA} \to 0})}} + {\Delta }\text{G}_{{{\text{sol(CNT} \to 0)}}} - {\Delta }\text{G}_{{{\text{sol(IMA - CNT} \to 0)}}}$$

∆G_sol(IMA →0)_, ∆G_sol(CNT→0),_ and ∆G_sol(IMA -CNT →0)_ denote free energy differences of disappearing IMA, CNT, and IMA - CNT complexes in solution.

## Results and discussion

In cancer therapy, a major challenge is to deliver anticancer drug molecules precisely to tumor sites for maximum treatment efficacy while minimizing side effects to normal organs. The development of advanced drug delivery systems shows great potential in improving the outcomes of cancer therapy. One approach is the creation of active targeting drug delivery systems, which utilize specific interactions between receptors on cell surfaces and targeting moieties attached to the surface of drug carriers. This enables the effective transportation of therapeutic drugs to tumor cells. However, challenges remain in improving the specificity and stability of drug delivery systems, regulating their bioavailability, and developing targeting carriers with lower toxicity. Therefore, the development of novel and effective drug delivery systems is of utmost importance.

Theoretical studies on drug delivery systems can provide valuable insights into the interactions between drugs and carriers, as well as aid in predicting drug release kinetics and optimizing formulations. However, the reproducibility of these theoretical findings in vivo remains a challenge. Animal models, particularly in the context of Parkinson's disease research, have been instrumental in elucidating pathogenic processes and developing new drug delivery systems [72]. Mathematical modeling has been employed in transdermal drug delivery to reduce the need for animal experiments and accelerate product design [73]. While theoretical studies offer a foundational understanding, the complexity of physiological environments and biological responses in animal models may not always align perfectly with theoretical predictions, highlighting the need for a multi-faceted approach combining theoretical insights with empirical validation in animal models.

Scaling up drug delivery systems for clinical use faces several challenges and limitations. These include issues related to controlling drug distribution and clearance in the blood, solubilizing poorly water-soluble agents, selectively targeting specific tissues, overcoming biological barriers, addressing drug bioavailability concerns, and synthesizing carriers with necessary properties [74]. Furthermore, the complexity of multifunctional drug carriers, difficulties in achieving controlled drug release, and challenges in fabricating carriers that meet all requirements hinder the implementation of stimuli-responsive systems for drug delivery [75, 76]. Overall, these factors collectively contribute to the limitations and challenges faced in scaling up drug delivery systems for clinical applications.

Molecular modeling studies have also been performed to gain a mechanistic understanding of surface functionalization of carbon nanotubes as a nanocarrier. DFT and MC simulation calculations were applied to investigate the drug delivery performance of the functionalized CNTs with carboxyl and hydroxyl groups in the gas phase and water solution. Functionalization of the surface of CNT can result in soluble materials, which can be further derivative with active molecules, making them compatible with biological systems.

Therefore, many biomedical applications can be envisaged. In other words, carboxylation and hydroxylation of CNT leads to an increase in solubility and decreases its toxicity and increase of drug absorption by nanotubes.

### QM

The research conducted here focused on investigating the interaction of a single drug molecule with the CNT surface, primarily due to computational constraints. The main objective was to explore the potential interaction between the drug and the nanotube serving as a carrier, while also identifying the key structural parameters influencing their properties. Computational analyses primarily operate at a microscopic scale, necessitating the determination of essential information including the CNT's functional group and drug loading capacities, as well as the intrinsic characteristics of both the drug and carrier for subsequent in vivo experimentation. Different synthesis methods impact the drug loading capacity and functional properties of CNTs. The scalability of the functionalization process of CNTs is influenced by several factors including strategies and methods used (such as physiochemical or acid treatment), practicality, achieved properties, endohedral or exohedral functionalization, emphasis on high performance for intended applications, impurities affecting yield, dispersion challenges in water or organic solvents, and difficulty in creating composites with other materials [77]. For instance, a facile one-step technology has been applied to produce functional carbon nanotubes in large quantities and various reactive groups (i.e., –OH, –NH2, –COOH, and –Br) were introduced onto the surfaces of CNTs without causing significant damage to nanotubes. The contents of the functional moieties can be easily controlled by adjusting the feed ratio [78].

**Fig. 2:**
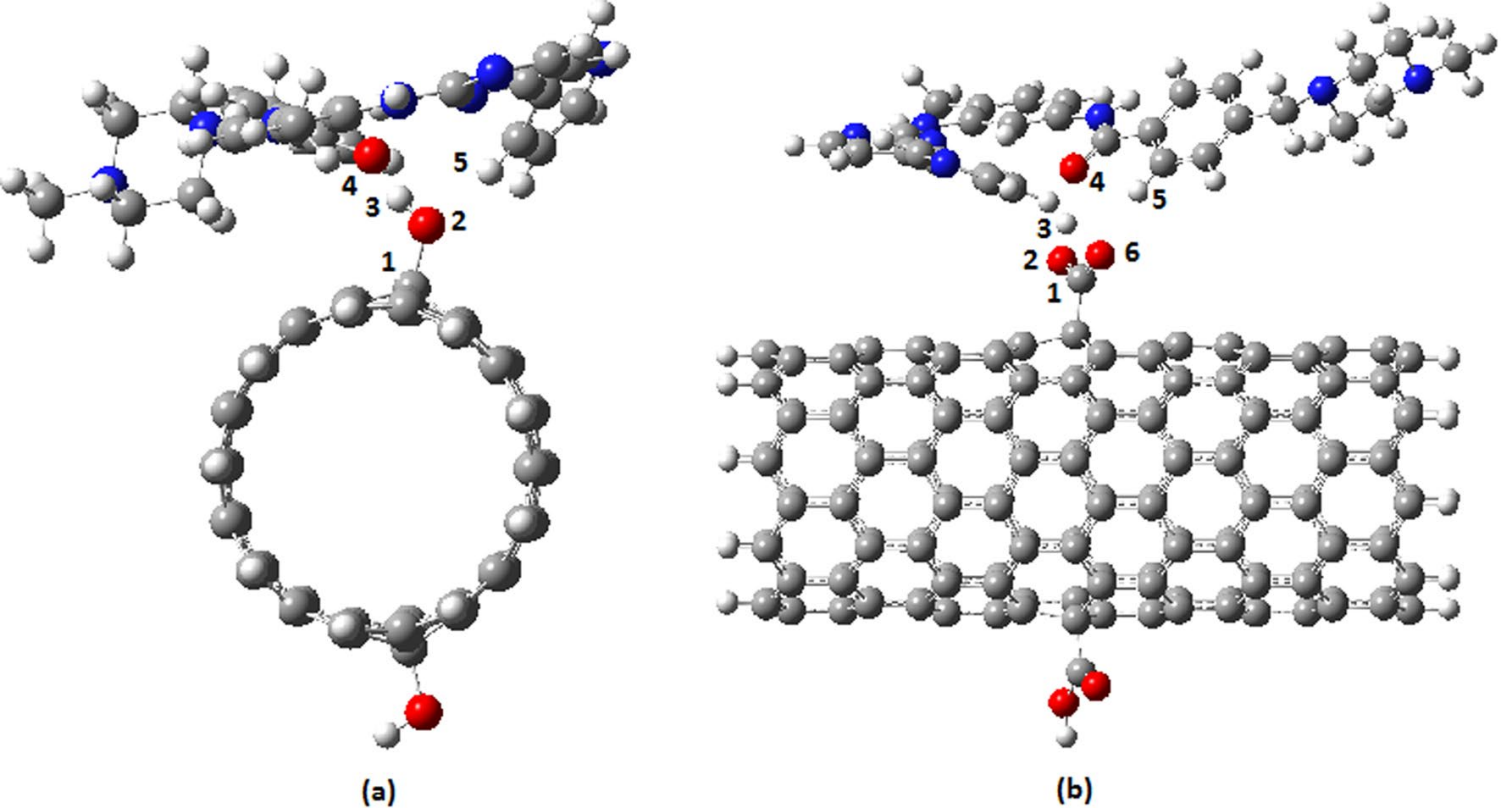
Optimized structures of **a** CNT-OH-D(CO) (top view) and **b** CNT-COOH-D(CO) side view

The QM section of our study aimed to compare the binding affinity of IMA with pure CNT and functionalized CNTs in the gas phase. As previously mentioned, the interactions between IMA and functionalized CNTs were explored through two potential binding sites, namely the pyridine ring and the carbonyl group. Additionally, the interaction between the drug and pure CNTs can occur through π–π stacking, which involves the aromatic rings of the loaded drug and the π-electrons on the surface of the CNT. This results in the formation of a non-covalently functionalized CNT-drug complex [79–81]. For example, the optimized structures of the IMA complexes (via CO) with OH and COOH functionalized CNTs are depicted in Fig. 2a and b respectively. Following optimization, the predominant orientations were determined based on the values of binding energies. The results of the quantum mechanical calculations on the studied models in the gas phase are presented in Table 1. The binding energy (E_b_) was calculated using the provided expression:

4 $${\text{E}}_{{\text{b}}} \left( {\text{BSSE corrected }} \right) = {\text{E}}({\text{CNT}} - {\text{IMA}}) - [{\text{E}}({\text{IMA}}) + {\text{E}}\left( {{\text{CNT}}} \right)] + {\text{BSSE}}$$

where E(CNT-IMA), E(IMA), and E(CNT)] determined the energy of optimized single structures of drug -CNT, drug, and CNT. Calculations were executed with counterpoise corrections for basis set superposition error (BSSE) [82].

**Table 1:** QM results in gas phase

Species	Dipole moment (Debye)	Binding energy (kcal /mol)	Bond length(Å)			
			[C-O4]	[C1-O2]	[C1-O6]	[O2-H3]
D(IMA)	5.39	–	1.228	–	–	–
CNT	0.00	–	–	–	–	–
CNT-OH	7.15	–	–	1.476		0.999
CNT-COOH	6.80	–	–	1.377	1.219	0.997
CNT-D	6.01	− 58.56	–	–	–	–
CNT-OH-D(CO)	7.14	− 26.27	1.258	1.463	–	1.016
CNT-COOH-D(CO)	12.37	− 16.45	1.2652	1.34	1.235	1.032
CNT-OH-D(PY)	10.05	− 14.51	–	1.446	–	1.033
CNT-COOH-D(PY)	13.76	− 14.48	–	1.326	1.237	1.109

The findings indicated that both CNT and functionalized CNTs can serve as adsorbents for the IMA drug. However, the binding energy for IMA adsorption on the outer surface of non-functionalized CNT is greater than that of functionalized CNTs in the gas phase. This outcome can be attributed to the presence of aromatic rings in the drug structure, which leads to adsorption on CNT through π–π interaction. Liu et al. [83] have also proposed that the non-covalent binding of doxorubicin to CNTs primarily occurs through supramolecular π–π stacking and hydrophobic interactions, owing to the aromatic nature of both DOX and the SWNT surface. The adsorption of ciprofloxacin on the outer surface of non-functionalized CNT has also been shown to occur through π–π stacking via their aromatic rings [84]. Furthermore, the utilization of non-functionalized CNTs is inappropriate for drug delivery due to the presence of impurities. Various impurities, including metal catalysts, amorphous carbon, and fullerenes, can be found on the synthesized CNTs [26]. To prevent any undesirable consequences, the materials employed as drug delivery systems should possess a high degree of purity.

In the case of functionalized CNTs, the drug is unable to approach the nanotube surface and establish π–π interaction. Instead, the main interaction between the drug and carrier involves hydrogen bonding between the hydroxyl or carboxyl group of functionalized CNTs (as either a donor or an acceptor hydrogen bond) and IMA. It has already been verified that hydrogen bonding plays a pivotal role in the interaction of doxorubicin with hydroxyl and carboxyl functionalized CNTs, as well as in the stability of the corresponding complexes [40]. Furthermore, a study on the interaction between carboxyl functionalized CNT and Droxidopa using the DFT method [85] demonstrated that the drug is adsorbed onto the CNT-COOH through hydrogen bonding and van der Waals interactions, thus confirming the physical nature of the adsorption.

In our study, the interaction between OH-functionalized CNT and IMA occurs through the CO group of the drug and the OH group of the CNT surface (Fig. 2a). However, this trend of binding energies will change in the solution phase. In the polar environment of aqueous solutions, hydrogen bonding, and dipole–dipole interactions are favored, making the interaction between functionalized CNTs and the drug more favorable. In fact, the main objective of the QM section was to precisely optimize the structures and investigate the potential interactions between the drug and CNTs.

Our results are also consistent with many other experimental or computational studies which validate the applied methodology. Adsorption of IMA molecule by a nanocomposite of CNT and metal–organic frameworks of Cu as an electrochemical sensor have been investigated [86]. The sensor exhibited very well operation toward the IMA and displayed acceptable reproducibility, reasonable repeatability, good stability, excellent sensitivity, and high selectivity for the detection of low amounts of the IMB in the biological and pharmaceutical samples. In another study, CNTs considerably improved the sensitivity and selectivity of the modified electrochemical sensor toward IMA [87]. IMA measurement has been also performed on the surface of modified N, S-doped CNT. The doped atoms with CNT expanded the effective surface area of the sensor, and the conductivity and wettability of the adsorbent improved [88]. DFT study of the adsorption of doxorubicin on hydroxyl/carboxylic functionalized SWNT and computed adsorption energy has confirmed that CNTs functionalized with carboxylic and hydroxyl groups have the potential to carry the drug [40]. DFT study (using wb97xd exchange–correlation functional and 6-311G(d) basis set) of the carboxylated CNT has indicated that it can be an efficient drug carrier for droxidopa drug by a binding energy of − 0.715 eV (− 16.49 kcal/mol) [85]. The adsorption mechanisms and interactions between the anticancer molecule Crizotinib on the surface of CNTs have been investigated using density functional calculations in both water and gas phases [89]. It has also been found that the adsorption energies of the complex drug-CNT in both gas and water had negative values, thus explaining the exothermic character of the interactions, and confirming CNT can act as a potential carrier for the Crizotinib. Four configurations of the interaction of drug and CNT have been investigated. The binding energy of the interactions like our study (parallel to the outer surface of pure CNT) was − 1.404 eV (32.38 kcal/mol) that are comparable with our results. Furthermore, it explored the aqueous solubility of the investigated complexes. The quantum mechanical computed solvation energy of CNT and CNT-drug in aqueous solution were − 24.96 and − 39.13 kcal/mol respectively which is comparable with our MC simulation results in water (discussed in section "MC"). The negative values for the solvation energy indicated that the solvation is spontaneous, thus demonstrating that the drug-nanotube complexes are soluble in water, which is a desirable factor for the use of the complexes as drug carriers in biological media.

An experimental study of the absorption of daunorubicin and etoposide by OH–and COO–functionalized CNTs for use in anticancer drug delivery has been conducted [90]. The absorption of etoposide and daunorubicin on pristine, hydroxylated or carboxylated nanotubes was investigated in vitro through the spectroscopy method. Experimental studies showed after 24 h the percentage of drug loading on OH- and COOH-functionalized nanotubes is somewhat larger than pristine CNT. The analysis showed that the functionalized CNTs are more suitable for the uptake of drugs and can be used for anti-cancer drug delivery. In another research, the interaction of the anticancer drug hydroxyurea with carboxyl functionalized CNT by employing DFT methods has been studied in gas and solvent phases [91]. The results show that all complexes are energetically favorable, especially in the aqueous phase. The adsorption energies of all considered configurations of the interaction of CNT-COOH and drug were in the range of − 5.1413 to − 13.6470 kcal/mol. The computed solvation energies were in the range of − 20.2060 to − 23.3636 kcal which are comparable with our outcomes. The kinetic and thermodynamic parameters have indicated that the drug could be adsorbed on the surface of functionalized CNT better than the pristine CNT.

The bond lengths involved in the interaction between IMA and CNTs are also provided in Table 1. As depicted in Table 1, the [C-O4] bond length of IMA is 1.228 Å, and after the interaction with CNT-OH and CNT-COOH, it will be 1.258 and 1.265 Å respectively. The interaction between the drug and CNT-OH and CNT-COOH (via CO) leads to binding via the O of the drug and the H of CNT-OH and CNT-COOH. Consequently, an increase in the length of the [C-O4] bond of IMA is anticipated after the interaction. Conversely, the participation of H3 of the [O2-H3] bond of the functionalized CNTs in hydrogen bonding with IMA results in a decrease in the length of the [C1-O2] bond upon interaction with the drug, followed by a slight increase in the [O2-H3] length (see Table 1). Similarly, the increase in the [C1-O6] bond length in CNT-COOH after interaction with IMA can be justified.

As observed, the percentage changes of OH bond [O2-H3] in CNT-OH and CNT-COOH before and after drug loading through CO are + 1.7% and + 3.5% respectively, which is compatible with the findings of the adsorption of Droxidopa drug onto carboxyl and hydroxyl functionalized CNTs [85]. Furthermore, the percentage changes of the CO [C1-O2] bond in CNT-OH and CNT-COOH before and after drug loading through CO were − 0.4% and − 0.37% respectively. It has been stated that during the adsorption process, the bond position remains unaltered, and only the bond length is affected by hydrogen bonding. In that study, the percentage change of the OH and CO bonds before and after drug adsorption was + 0.4 and − 0.7 respectively. Therefore, it is evident that after the drug loading on the CNT surface, no significant changes were observed in the structure of IMA, thereby preserving its properties. Consequently, functionalized CNTs can serve as drug carriers and safeguard the drug from degradation.

The release of the drug from the carrier is a crucial step in the drug delivery process. It is important to note that strong interactions between the carrier and drug are unfavorable in a drug delivery system, as they can make it difficult for the drug to be released and result in long recovery times for the carrier. If the adsorption energy (E_ad_) is significantly increased, the drug release time becomes much longer. To detach the drug from the carrier, either external stimuli or internal stimuli operating within a biologically controlled can be used. The recovery process is achieved by heating the adsorbent to higher temperatures or exposing it to light [92]. When the drug-carrier is exposed to light, a process of drug desorption may occur. The duration of drug desorption corresponds to the recovery time of the carrier, which can be calculated using the conventional transition state theory [93]. Using transition state theory, the recovery time τ from the carrier surfaces relates to the adsorption energy, E_b_,

5 $$\tau ={\nu }_{0}^{-1}{e}^{(-{E}_{b}/{k}_{b}T)}$$

where T is the temperature of the system, k_b_ the Boltzmann’s constant, and υ_0_ an attempt frequency. Equation (5) indicates that a larger E_b_ value results in a longer recovery time. If the drug desorption from the CNT surfaces takes too long, it is not suitable for an effective drug delivery process. Molecules have characteristic absorption frequencies that depend on rotational and vibrational energy transitions, as well as temperature. For example, the CO2 molecule has an attempt frequency of 10^13^ Hz, so light with a frequency of 10^12^ Hz should be applied for NO2 [94].

According to the equation, more negative E_ad_ values will exponentially prolong the recovery time. The times for the desorption process of IMA molecule from the CNT surfaces at 298 K are given in Table 2. As can be seen, if an attempt frequency of υ_0_ of 10^12^ Hz is applied, the recovery time of IMA molecules from the CNT-OH surface (via CO) is 214.4 days while releasing from CNT-COOH takes 1.2 s. However, the recovery time for the binding through PY from both CNT-OH and CNT-COOH is about 40 s. Overall, the release of the drug from CNT-COOH is much faster than from CNT-OH due to its higher affinity with CNT-OH. The results are comparable to the recovery time of Pramipexole molecules from the gold surfaces at 298 K which have been in the range of 1.2 ms to about 10^5^ s [95]. In another study the recovery time for the adrucil/Al-doped phagraphene complex was approximately 4.09 × 10^18^ s at 298 K. This indicates that the Al-doped phagraphene has a very long recovery time and cannot be used as a drug carrier [96]. The calculated recovery time values for 6-TG desorption from metalloborospherenes were also obtained to be about 10^−12^ s [97], indicating that 6-TG adsorption to the carriers is reversible.

**Table 2:** The recovery time of IMA from the CNTs at 298 K

ν(Hz)	Recovery time			
	CNT-OH-D(CO)	CNT-COOH-D(CO)	CNT-OH-D(PY)	CNT-COOH-D(PY)
10^12^	214.4 day	1.2 s	43.9 ms	41.7 ms
10^13^	21.4 day	0.12 s	4.4 ms	4.2 ms
10^14^	51.4 h	11.6 ms	0.4 ms	0.4 ms

To gain a deeper insight into how COOH and OH functionalization influences the properties of CNTs and their performance as a drug delivery system the changes in partial atomic charges and energies of frontier orbitals after functionalization were analyzed. The Mulliken atomic charges of selected IMA and CNT atoms are presented in Table 3. The computed QM atomic charges were employed to calculate intermolecular electrostatic interactions in subsequent MC simulation computations. Furthermore, comparing the atomic charges before and after the interaction of IMA and CNT indicates the charge transfer between the drug and nanotube, thereby confirming the interaction. The comparison of the atomic charges at the site of interactions indicates the effectiveness of that site. As can be seen, the oxygen (O4) and pyridinic nitrogen atomic charge of the drug in the complexes of functionalized CNTs are larger than nonfunctionalized CNTs leading to more intermolecular electrostatic interactions in aqueous solution. Moreover, the atomic charges of functional groups (O2, C1, H3, and O6) induced improvement of the solubility of CNT. It is anticipated that incorporating hydrophilic groups such as carboxylic or hydroxyl groups onto the external walls of CNTs will enhance the dispersibility in aqueous solutions and enhance their biocompatibility.

**Table 3:** Selected Mulliken atomic charges of IMA and CNT complexes

Species	q(O4)	q(O2)	q(C1)	q(H3)	q(O6)	q(N)
D(IMA)	− 0.821	–	–	–	–	− 0.640
CNT-OH	–	0.583	0.137	0.314	–	–
CNT-COOH	–	− 0.634	1.029	0.375	− 0.738	–
CNT-D	− 0.839	–	–	–	–	− 0.640
CNT-OH-D(CO)	− 0.906	− 0.564	0.159	0.425	–	− 0.646
CNT-COOH-D(CO)	− 0.959	− 0.657	1.08	0.469	–0.803	− 0.612
CNT-OH-D(PY)	− 0.890	− 0.643	0.178	0.430	–	− 0.751
CNT-COOH-D(PY)	− 0.829	− 0.724	1.060	0.486	− 0.773	− 0.800

To evaluate the effect of COOH and OH functional groups on the reactivity of CNT, further examine the energies of frontier orbitals were applied. The highest occupied energy (HOMO), lowest unoccupied energy (LUMO), HOMO–LUMO band gap (E_g_), and change of band gap of CNT(∆E_g_) after functionalization and drug adsorption were calculated and indicated in Table 4. According to the data presented in Table 4, the band gap of functionalized CNTs with OH and COOH is approximately 15% and 12% lower, respectively, compared to pristine CNTs. The relatively larger HOMO–LUMO gap of CNTs correlates with their reduced reactivity towards drugs in comparison to functionalized CNTs. The results indicate that the interaction between drugs and functionalized CNTs increases the band gap by an average of 5%. These results were compatible with the earlier study that has revealed by creating stable complexes with drugs, such as fluorouracil, functionalized CNTs can activate them, thus potentially decreasing side effects and improving bioavailability [98].

**Table 4:** Energies (eV) of frontier orbitals, HOMO–LUMO band gap (Eg) and change of Eg (upon functionalization and IMA adsorption of CNT)

Species	HOMO	LUMO	Eg	ΔEg%
D(drug)	− 0.2729	0.0072	0.2801	–
CNT	− 0.1839	− 0.0962	0.0877	–
CNT-OH	− 0.1866	− 0.1121	0.0745	− 15.05
CNT-COOH	− 0.1869	− 0.1101	0.0768	− 12.44
CNT-D	− 0.1893	− 0.0966	0.0927	+ 5.74
CNT-OH-D(CO)	− 0.1883	− 0.1098	0.0785	+ 5.38
CNT-COOH-D(CO)	− 0.1864	− 0.1064	0.0800	+ 4.21
CNT-OH-D(PY)	− 0.1848	− 0.1071	0.0777	+ 4.36
CNT-COOH-D(PY)	− 0.1830	− 0.1015	0.0816	+ 6.28

The bonding mechanism between drugs and CNTs can be elucidated through the analysis of frontier orbitals. The HOMO and LUMO states of both the drug and CNTs play a crucial role in these interactions. Depending on the energy gap, either forward or backward donation dominates. The energy difference between the HOMO of IMA and the LUMO of CNT is 0.18 eV, which is smaller than the value of 0.19 eV between the HOMO of CNT and the LUMO of IMA. Therefore, the forward donation (IMA/CNT) is expected to be more effective, and this interaction is primarily characterized by electron transfer through π → π* interaction from IMA to CNT. In other words, CNTs tend to act as electron acceptors, while the drug acts as an electron donor. In the case of the interaction between functionalized CNTs and the drug, the energy differences between the HOMO of functionalized CNTs (CNT-OH and CNT-COOH) and the LUMO of the drug are approximately 0.19 eV, which is greater than the differences between the HOMO of the drug and the LUMO of functionalized CNTs (0.16). Consequently, electron transfer from the HOMO of the drug to the LUMO of CNTs occurs more easily, indicating that the drug tends to act as a donor, while functionalized CNTs act as acceptors. Hence, the primary interaction between functionalized CNTs and the drug occurs through electron transfer from the drug to COOH– or OH-functionalized CNTs, which exhibits a hydrogen bond nature.

Since an overlap between the frontier orbitals of CNTs and IMA molecule is normally followed by a charge transfer, further examine the NBO charge distribution of systems was applied to gain more insight into the problem. To examine the interaction among bonds, the natural bond orbital (NBO) parameters for the interactions of IMA-CNT complexes were calculated, and the obtained results are presented in Table 5. The stabilization energy, E2 (second-order perturbation energy), associated with the donor orbital (i) and acceptor orbital (j), E(j) and E(i) represent orbital energies, while Fij denotes the off-diagonal NBO Fock matrix element j. The elucidation of the nature of the interaction between the electron of the donor orbital and the electron of the acceptor orbital, as well as the determination of the conjugation extent of the entire system, are both revealed by the value of E(2).

**Table 5:** The Second-order Perturbation Energies for CNT-IMA, CNT-OH-IMA, and CNT-COOH-IMA intermolecular interactions

Species	Donor	Acceptor	E(2) (kcal/ mol)	E(j)-E(i)(a.u.)	F(i,j)(a.u.)
CNT-D	π (C–C) **(D)**	π*(C–C) **(CNT)**	0.15	0.36	0.007
CNT-OH-D(CO)	LP(O4) **(D)**	σ* (H3) **(CNT)**	26.80	0.62	0.127
	LP(O2) **(CNT)**	σ* (C-H5) **(D)**	6.32	0.99	0.071
CNT-COOH-D(CO)	LP(O6)** (CNT)**	σ* (C-H5) **(D)**	3.83	0.96	0.056
	LP(O4) **(D)**	σ* (H3) **(CNT)**	36.32	0.54	0.126
CNT-OH-D(PY)	LP(N)(PY) **(D)**	σ* (H3) **(CNT)**	57.05	0.66	0.191
CNT-COOH-D(PY)	LP(N)(PY) **(D)**	σ* (H3) **(CNT)**	33.64	0.61	0.275
	LP(O6) **(CNT)**	σ* (C-H) **(D)**	4.41	0.93	0.059

The findings from Table 5 demonstrate that the most powerful intermolecular interaction between IMA and CNT-OH, and CNT-COOH, through the CO group, occurs via electron transition from LP(O4) → σ* (H3) with E(2) values of 26.80 and 36.32 kcal mol^−1^ respectively. This result confirms the presence of hydrogen bonding, where the electron transition takes place from the lone pair of O(CO) in the drug to the hydrogen of the COOH or OH functional groups in CNT. Similarly, the strongest intermolecular interaction between IMA and, CNT-OH and CNT-COOH, through pyridine, occurs via electron transition from LP(N) → σ* (H3) with E(2) values of 57.05 and 33.64 kcal mol^−1^ respectively. This also indicates the presence of hydrogen bonding, with the electron transition from the lone pair of N(pyridine) in the drug to the hydrogen of the COOH or OH functional groups in CNT. However, the calculated binding energies show that IMA exhibits the strongest binding with non-functionalized CNT. The results from Table 2 reveal that this interaction occurs via π (IMA) → π*(CNT) transition, with a lower E(2) value compared to the other interactions. The strength of this interaction is attributed to a significant number of π (IMA) → π*(CNT) transitions through π-π stacking. These results align with the calculated binding energies.

As shown in Table 1, the dipole moments of all D-CNT complexes are greater than that of IMA. Therefore, it can be predicted that the solubility of D-CNT complexes in aqueous solution is higher than that of IMA. This implies that the transport of the drug in physiological media is facilitated. The utilization of these functionalized CNTs not only enhances bioavailability by increasing the drug's solubility in an aqueous environment but also promotes the appropriate interaction of these functionalized CNTs with the IMA. This interaction results in stability and efficient transport in the physiological environment, which can be compared to previously studied carriers for IMA. A calixarene-based drug carrier was specifically designed to transport IMA [99], displaying an interaction energy value below 13.84 kcal/mol as determined through DFT computations at the B97D/6-31G(d,p) level of theory. The investigation into utilizing metal–organic frameworks as a potential drug delivery system for IMA [100] revealed that the binding energies of MIL-101(Cr) and MIL-100(Fe) towards IMA were 9.90 and 9.17 kcal mol^−1^, respectively. A comparison of the interaction strengths between IMA and functionalized CNTs with those carriers suggested that imatinib would be more readily and strongly adsorbed into functionalized CNTs.

In the next part, the solvation behavior of these nano drug complexes as well as the possibility of interactions between IMA and functionalized and non-functionalized CNTs in aqueous solution is evaluated using MC Simulation.

### MC

In the QM part, the probability of the interaction between IMA and CNTs was investigated. Initially, the total interaction energies of the structures (CNT, CNT-OH, CNT-COOH, IMA, and IMA-CNT complexes) were determined through MC simulations to assess the interaction between IMA and CNTs in an aqueous solution.

The total energy (E_tot_) in solution is described as the sum of contributions from solute-water, water-water, and intramolecular interaction energies.

The energies obtained from the MC simulation are presented in Table 6. This table includes the number of water molecules in the box (N_H2O_), the energy contributions of solute-H2O interaction energy (E_soln_), as well as the electrostatic contribution and the van der Waals contribution to the solute-H2O interaction energy.

**Table 6:** MC simulation results (energies are in kcal/mol)

Structure	N_H2O_	E(H2O-solute)	E(total)	Van der Waals contribution in E(H_2_O- solute)	Electrostatic contribution in E(H_2_O- solute)
D(drug)	4049	− 2.72	− 11.47	− 0.085	− 2.633
CNT	4009	− 9.83	− 9.75	− 0.43	− 0.635
CNT-OH	4010	− 15.14	− 18.76	− 0.37	− 14.78
CNT-COOH	4006	− 14.51	− 18.32	− 0.38	− 14.13
CNT-D	3992	− 10.08	− 15.46	− 0.38	− 9.69
CNT-OH-D(CO)	3978	− 15.33	− 19.04	− 0.37	− 14.96
CNT-COOH-D(CO)	9380	− 14.96	− 18.85	− 0.41	− 14.55
CNT-OH-D(PY)	3993	− 8.07	− 14.18	− 0.37	− 7.69
CNT-COOH-D(PY)	3973	− 7.63	− 13.94	− 0.41	− 7.22

Total energies of functionalized-CNTs (CNT-OH and CNT-COOH) in solution were found to be higher than that of non-functionalized CNT due to the larger electrostatic contribution in solute-H2O interaction energies of CNT-OH and CNT-COOH. The functionalization of CNT leads to an increase in partial atomic charges on the tube wall due to charge transfer between CNT and functional groups. As a result, electrostatic interactions between CNT and solvent are enhanced. These findings align with the experimental characterization of COOH functionalized CNT conducted by Muratov et al. [101]. Their study demonstrated that the CNT surface becomes hydrophilic after chemical modification with COOH. The COOH functionalized CNTs exhibited stable water dispersions and showed great potential for biological applications.

The total energies of complexes formed between functionalized carbon CNTs and IMA[CNT-OH-D(CO), CNT-COOH-D(CO)] in solution were found to be greater than those of non-functionalized CNT-IM(CNT-D) complex. Additionally, Monte Carlo simulations revealed that the total energy of IMA alone was − 11.47, which was lower than that of the CNT-IMA complexes. The interaction between IMA and CNTs led to an increase in the electrostatic contribution to the solute-water interaction energy, consequently resulting in an elevation of the total interaction energy of IMA. This interaction between IMA and the nanotube also led to an enhancement of partial atomic charges, thereby amplifying the electrostatic contribution to the IMA-water interaction energy. Furthermore, the electrostatic contribution to the CNT-IMA interaction energy was found to be larger than the van der Waals contribution. The partial atomic charges had a direct impact on the electrostatic terms of intermolecular energies. In aqueous solutions containing IMA-CNT complexes, electrostatic interactions played a predominant role in intermolecular interactions. Conversely, the role of van der Waals interactions in the intermolecular interactions of CNTs and CNT-IMA solutions was nearly identical. Thus, it can be concluded that van der Waals interactions do not significantly affect the intermolecular energies following the interaction of IMA and CNTs in water. These findings align with our previous research [102]. Similarly, a simulation study involving cisplatin encapsulated in nanotubes demonstrated an increase in electrostatic interaction energies after the drug and CNTs interacted, while the van der Waals energies remained unchanged [102]. Encapsulation of alkylating anticancer drugs within CNTs also increased the electrostatic and total interaction energies but did not affect the van der Waals interactions in solution [103].

Enhancing the solubility of IMA from its dosage form is of great importance for its in vivo bioavailability and therapeutic effectiveness. The solvation free energies of CNTs, IMA, and IMA-CNT complexes were determined using Eq. 2, and the computed solvation free energies are presented in Table 4. As evident, the solvation free energy of IMA significantly increased after interacting with both functionalized and non-functionalized CNTs. The solvation free energies of CNT-IMA complexes are expected to be substantial due to the significant contribution of electrostatic interactions in the IMA-CNT complexes to the solute–solvent interactions (Table 6). While the solvation free energy of IMA alone was approximately − 27.21 kcal mol^−1^, primarily due to the small electrostatic contribution to the drug-solvent interaction energy (− 2.633 kcal mol^−1^), it can be predicted that when IMA is attached to CNTs (non-functionalized CNT, CNT-OH, CNT-COOH), the resulting complexes would be more stable in water than the drug alone, thereby increasing the likelihood of a reaction in an aqueous environment.

Furthermore, to predict the likelihood of the interaction between CNTs and IMA in an aqueous environment, the association free energies were calculated. As shown in Table 7, the association free energy of the interaction between all functionalized CNTs (CNT-OH, CNT-COOH) and IMA via both sites was negative, whereas the association free energy of the interaction between non-functionalized CNTs was positive. Therefore, only functionalized CNTs can interact with this drug in an aqueous solution. The non-spontaneous interaction between IMA and nonfunctional CNT in a solution is attributed to the limited solvation of CNT and IMA in water environments.

**Table 7:** Solvation free energies (ΔG_sol_) and association free energies (ΔG_ass_) of drugs–CNT compounds

Species	ΔG(sol) (kcal/mol)	ΔG(ass) (kcal/mol)
D(drug)	− 27.21	–
CNT	− 8.882	–
CNT-OH	− 109.38	–
CNT-COOH	− 104.49	–
CNT-D	− 73.53	37.438
CNT-OH-D-CO	− 114.62	− 21.97
CNT-COOH-D-CO	− 119.77	− 11.93
CNT-OH-D-PY	− 60.25	− 76.34
CNT-COOH-D-PY	− 56.24	− 75.46

In order to determine the distribution of water molecules inside and outside the CNT, radial distribution functions (RDFs) were calculated. RDF is a characteristic that describes the structure of fluids. It is calculated as the ratio of the local density ($\rho (r)$) to the bulk density. The RDF is a function of the distance from the central axis of the nanotube, $g\left(r\right),$

6 $$g\left(r\right)=\frac{\rho \left(r\right)}{{\rho }_{bulk}}$$

**Fig. 3:**
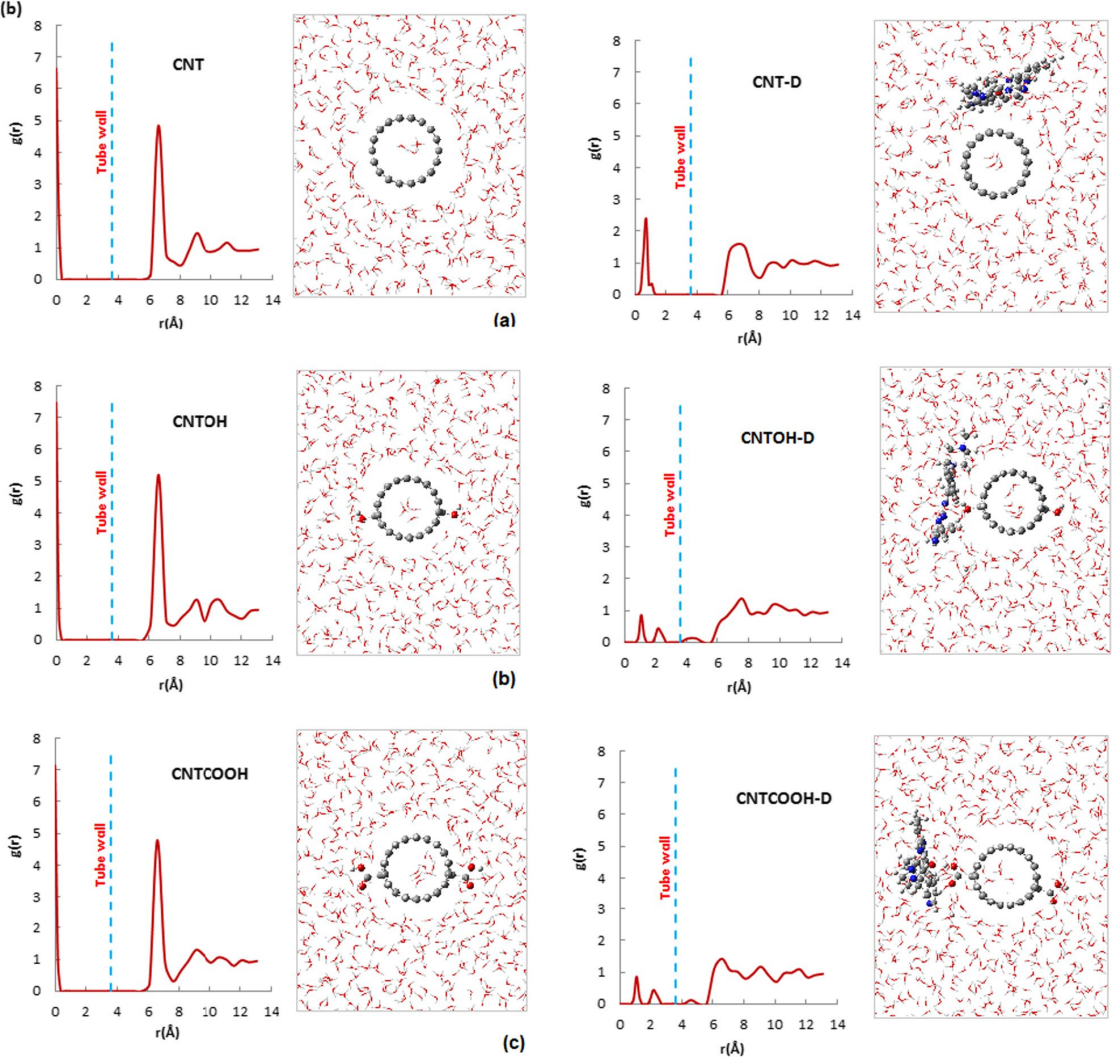
RDFs of water molecules from center of nanotube and Snapshot of the simulation of **a** non-functionalized CNT and CNT-IMA complex **b** CNTOH and CNTOH-IMA complex **c** CNTCOOH and CNTCOOH-IMA complex

Figure 3 illustrates the RDF diagrams for the CNTs and their complexes with IMA. These diagrams depict g(r), which represents the distribution of water molecules inside and outside the CNT as a function of the distance (r) from the axis of the nanotube. The inner and outer plots are separated by a dashed line located at 3.61 Å (the radius of the CNT). The inner peaks of the RDF plots for the CNTs are primarily located at the center of the nanotube (0–1 Å from the central axis), whereas for the complexes, they are shifted towards the tube wall. This shift is attributed to an increase in the hydrophilicity of the tube wall. The sharp and high peak at the center of the CNT indicates the accumulation of water molecules in the center of the nanotube due to the hydrophobic nature of the tube wall.

Furthermore, the RDF graphs for distances outside the CNT (r > 3.61 Å) exhibit peaks that correspond to the formation of shell-like water molecules around the CNT surface.

In the RDF graph of pure CNT (Figure (a)), the first and highest peak is observed at 6 Å (2.33 from the outer surface of the nanotube), and subsequent layers with lower intensity are located around 9 and 11 Å. However, in Fig. 3, it can be observed that for all three CNT-IMA complexes (CNT-D, CNT-OH-D, CNT-COOH-D), the intensity of all peaks has decreased, and they have become broader. This can be attributed to the interaction between water molecules and the drug molecule that is bonded to the surface of the nanotube, as well as the interaction between water molecules themselves. In fact, the interaction between IMA and CNT leads to water molecules within the shells pointing towards each other, resulting in broader peaks compared to pure CNT. Additionally, the RDF diagrams for the CNT-OH-D and CNT-COOH-D complexes exhibit a small peak near the outer wall of the nanotube, indicating the density of water molecules around the carboxyl and hydroxyl groups and the hydrophilicity of these functional groups on the CNT.

It is worth noting that all RDF plots demonstrate convergence to the bulk water limit at distances greater than 12 Å (approximately 8.4 Å from the nanotube surface), which is consistent with our previous research [102, 103].

Figure 3 also presents snapshots of configurations extracted from Monte Carlo simulations of non-functionalized CNT, functionalized CNTs with hydroxyl and carboxyl groups, and their complexes with IMA in an aqueous solution. The figures compare the qualitative scheme of the hydration shells surrounding the CNT.

The main objective of the current study was to evaluate the efficiency of carboxyl and hydroxyl functionalized CNTs as a drug delivery system. The efficiency of drug delivery systems depends on many parameters, including stability in the bloodstream, targeting the desired tissues, uptake by the target cells, and final release of the drug. Computational studies can characterize important aspects of a drug delivery system, regarding drug loading, the stability of complexes, the interactions with membranes, and drug release.

To evaluate the nanocarrier as a potential drug delivery system, the carrier should be a good adsorbent for binding with the drug as well as a good drug carrier in physiological media and must have a good performance to release the drug in target cells. Therefore, the binding energies of the drug and functionalized CNTs as adsorbent were evaluated, and then interactions of drug and CNTs as drug transporters in an aqueous solution were assessed in terms of association free energy. Moreover, to serve the biocompatibility of these nano-drug compounds, the solvation of CNT-IMA complexes had to be evaluated. The negative values of the adsorption and solvation energies demonstrated that the CNT-drug complexes are stable, the interaction of the drug molecule with the nanotube sidewall is a spontaneous process and the CNT can act as a potent carrier for IMA. This carrier system was suitable as a drug delivery system when could release the drug in target cells. Good adsorption on any surface does not indicate a capable drug carrier since the release will not easily be done.

As it was mentioned all binding energy of CNT, CNT-OH, and CNT-COOH with IMA molecule were negative (Eb < 0) indicating the bindings were possible in the gas phase. The computed solvation free energies confirmed the solubility and biocompatibility of the IMA-CNT complexes however, only functionalized CNTs have a negative association free energy with IMA in an aqueous environment, and therefore CNT-OH and CNT-COOH can act as an efficient drug adsorbent and transporter in physiological media.

Based on advanced chemical alterations, the key factor essential for the effective practical application of CNTs as carriers of pharmaceutical agents lies in comprehending the underlying mechanisms responsible for their pharmacological and toxicological impacts. The precise evaluation of pharmacological parameters, including the absorption, transportation, target delivery effects, blood circulation time, clearance half-life, organ biodistribution, and accumulation, are essential prerequisites for them to be developed into practically usable drugs [104]. In the context of utilizing CNTs as drug carriers, their uptake from the site of administration into the organism is a critical requirement. Subsequently, the transported CNTs need to reach the relevant areas of effect, such as tumor sites, infection sites, and ischemic regions, among others. Following administration, absorption stands as the primary pivotal stage for drug carriers to fulfill their task of delivering pharmaceuticals. As studied in current research (and many earlier studies) research transporting capabilities of carbon nanotubes combined with appropriate surface modifications and their unique physicochemical properties show great promise to sufficiently strong adsorptive effects for anticancer drugs to ensure they can transport the drugs to the effect-relevant sites, and they can release the drugs from them in the effect-relevant sites. Certain drugs encounter challenges in reaching cancerous tissues due to their short residence time in blood circulation. The prepared CNT-drug complexes had longer blood circulation time and higher tumor-specific drug accumulation. Moreover, the widely used chemotherapeutic agent in cancer therapy is often limited by its poor solubility in aqueous medium and nonspecific cytotoxicity, thereby preventing it from efficiently reaching the cancer focuses. As a result of current research by using the functionalized CNT as a drug delivery system, the solubility of IMA has been enhanced. Some other groups have also developed drug-loaded CNTs that were internalized efficiently by cancer cells, leading to subsequent drug release and translocation to the cell nucleus, CNTs remained in the cytoplasm [31]. At acidic pH, hydrophilicity of drug is enhanced, which facilitates its detachment from the CNT surface. The intelligent release of drugs within tumor tissues has been demonstrated through such systems, taking advantage of the acidic conditions present in the tumor environment and intracellular lysosomes.

Many types of cancer spread through the lymphatic system, and drug delivery systems tailored for this purpose can effectively impede cancer metastasis. The absorption of bundled CNTs by the lymphatic system appears to be facilitated by the larger fenestra present in lymphatic capillary endothelial cells compared to blood capillary endothelial cells [12]. The lymphatically absorbed CNTs migrate along the lymph canal and accumulate in the lymph node, which is in fact a lymphatic target effect. This is clinically important because lymphatic metastasis occurs extensively in cancers, resulting in frequent tumor recurrence, even after extended lymph node dissection. If anticancer drugs are loaded into CNTs, they will be delivered into the lymph system, where the drugs will be released to kill metastatic cancer cells.

Surface-modified CNTs can be functionalized by targeting ligands to achieve targeted drug delivery, directing therapeutic agents to specific sites in the body for enhanced efficacy. CNTs enable target drug delivery through their unique properties, facilitating precise transport of therapeutic agents to specific sites, and enhancing pharmacological efficacy in drug delivery systems [105]. CNTs enable targeted drug delivery through precise control of the nano-pumping process using external heat flux and atomic defects, enhancing hydrodynamic and thermal performances for clinical applications [106]. The paper has discussed the use of CNTs for targeted drug delivery through lung cancer cells, emphasizing the importance of systemic delivery design. Target drug delivery with CNTs has been achieved through functionalization, trajectory manipulation, and force control, enhancing precision and efficacy in reaching specific areas within lung cancer cells [107]. The study has designed a carrier system for CNTs to cross the lung cell membrane within 30 s to 5 min and achieve a translocation profile of 1 to 100 Å for effective drug delivery. The study proposed a delivery system for CNTs to effectively cross the lung cell membrane and achieve a specific translocation profile for targeted drug delivery. Another Research findings highlight the efficacy and safety of CNT approaches for treating various diseases such as brain tumors, cancer, and other biomedical applications through nanotechnology-based drug delivery systems [108]. Additionally, the conjugation of CNTs with various compounds like polysaccharides, proteins, and magnetic nanoparticles allows for smart targeting and trajectory manipulation, essential for successful drug delivery in fields such as cancer diagnostics and treatments [109]. These approaches collectively contribute to the advancement of targeted drug delivery using CNTs from a pharmacological perspective.

## Conclusions

The side effects associated with pharmaceutical drugs present a significant challenge in the field of medicinal chemistry. To the management of IMA drug side effects, CNT was applied as a drug carrier, and to improve the solubility, biocompatibility, and drug-carrying capacity of CNT, the process of functionalization by carboxyl (COOH) and hydroxyl (OH) groups was employed in this study. The initial section of this research involved the calculation of binding energies between the IMA drug and both pure and functionalized CNTs applying density functional calculations. The findings demonstrated that in the gas phase, the IMA drug can adsorb onto the surfaces of both pure and functionalized CNTs through various interactions involving the pyridine ring and carbonyl group. To investigate intermolecular bonding, NBO analysis was applied to the interactions of IMA-CNT complexes. The strongest IMA-CNT intermolecular interaction occurred via oxygen of carbonyl group in the drug to the hydrogen of the COOH or OH functional groups in CNT (interaction through carbonyl group), and electron transition from the lone pair of N in the drug to the hydrogen of the COOH or OH functional groups in CNT(interaction through pyridine group).

In the second section, the stability of drug-CNT complexes in an aqueous solution was estimated by Monte Carlo simulation and free energy perturbation method. Computational analysis indicated that the solvation free energies of all IMA-CNT complexes in water were significantly larger compared to IMA alone. However, it was observed that only the COOH and OH functionalized CNTs were able to interact with IMA in the aqueous solution. The calculated association free energies revealed that there is no possibility of interaction between non-functionalized CNT and IMA in water media.

As a result, it can be predicted that carboxyl and hydroxyl functionalized CNT have the potential to serve as effective carriers for IMA in an aqueous solution with minimal drug conformational changes as well as improved solubility of IMA in biological fluids to enhance its bioavailability. Although this research has computationally demonstrated the efficacy of these functionalized CNTs, further investigations need to be conducted in vitro or in vivo to advance these CNTs toward future clinical trials. The prospective studies should primarily concentrate on two aspects. Firstly, it is imperative to rationally design and fabricate alternative CNTs with other functional groups and examine the interactions between these nanoscale platforms and various biological entities and drugs. Secondly, it is crucial to explore the pharmacokinetics and biocompatibility of these nanocarriers and investigate their toxicity on diseased tissues as well as their side effects on healthy tissues in vivo. Although there are still numerous unresolved questions regarding the practical clinical applications of these nanomaterials as drug delivery systems, encompassing synthesis, transportation, and drug release, it is anticipated that these issues will be resolved in the future.

## Data Availability

The optimized geometries of the molecules, and datasets generated and analyzed during the current study are available from the corresponding author upon request.
